# The AP-1 Transcription Factor c-Jun Prevents Stress-Imposed Maladaptive Remodeling of the Heart

**DOI:** 10.1371/journal.pone.0073294

**Published:** 2013-09-10

**Authors:** Renata Windak, Julius Müller, Allison Felley, Alexander Akhmedov, Erwin F. Wagner, Thierry Pedrazzini, Grzegorz Sumara, Romeo Ricci

**Affiliations:** 1 Institute of Cell Biology, Eidgenössische Technische Hochschule Zurich (ETHZ), Zurich, Switzerland; 2 Experimental Cardiology Unit, Department of Medicine, University of Lausanne Medical School, Lausanne, Switzerland; 3 Cardiovascular Research, Institute of Physiology, University of Zurich, Zurich, Switzerland; 4 Genes, Development and Disease Group, F-BBVA Cancer Cell Biology Programme, Spanish National Cancer Research Centre (CNIO), Madrid, Spain; 5 Institut de Génétique et de Biologie Moléculaire et Cellulaire (IGBMC), Institut National de la Santé et de la Recherche Médicale, Centre National de la Recherche Scientifique, Université de Strasbourg, Illkirch, France; 6 Laboratoire de Biochimie et de Biologie Moléculaire, Nouvel Hôpital Civil, Hôpitaux Universitaires de Strasbourg, Université de Strasbourg, Strasbourg, France; Albert Einstein College of Medicine, United States of America

## Abstract

Systemic hypertension increases cardiac workload and subsequently induces signaling networks in heart that underlie myocyte growth (hypertrophic response) through expansion of sarcomeres with the aim to increase contractility. However, conditions of increased workload can induce both adaptive and maladaptive growth of heart muscle. Previous studies implicate two members of the AP-1 transcription factor family, junD and fra-1, in regulation of heart growth during hypertrophic response. In this study, we investigate the function of the AP-1 transcription factors, c-jun and c-fos, in heart growth. Using pressure overload-induced cardiac hypertrophy in mice and targeted deletion of *Jun* or *Fos* in cardiomyocytes, we show that c-jun is required for adaptive cardiac hypertrophy, while c-fos is dispensable in this context. c-jun promotes expression of sarcomere proteins and suppresses expression of extracellular matrix proteins. Capacity of cardiac muscle to contract depends on organization of principal thick and thin filaments, myosin and actin, within the sarcomere. In line with decreased expression of sarcomere-associated proteins, *Jun*-deficient cardiomyocytes present disarrangement of filaments in sarcomeres and actin cytoskeleton disorganization. Moreover, *Jun*-deficient hearts subjected to pressure overload display pronounced fibrosis and increased myocyte apoptosis finally resulting in dilated cardiomyopathy. In conclusion, c-jun but not c-fos is required to induce a transcriptional program aimed at adapting heart growth upon increased workload.

## Introduction

Systemic arterial hypertension, valvular pathologies as well as myocardial infarction represent exceedingly frequent clinical manifestations contributing to heart failure and/or malignant ventricular arrhythmias [1]. Initially, these abnormalities and associated mechanical stress as well as hormonal changes induce an orchestrated signaling network in the heart that underlies myocyte growth through expansion of sarcomeres to increase contractility as well as activation of stress and fetal gene programs. In the long term, however, these mechanisms may trigger pathologic remodeling of the myocardium ultimately leading to heart dilation and reduced cardiac performance [2], [3]. Cardiomyocyte apoptosis/necrosis and progressive interstitial fibrosis are hallmarks that contribute to ventricular wall thinning and chamber dilation [4]. The latter alterations are also frequently found in dilated and hypertrophic cardiomyopathies of genetic origin that are caused by mutations in sarcomeric genes that constitute the contractile apparatus of cardiac muscle cells [5].

The Mitogen-Activated-Protein-Kinase (MAPK) family represents an essential signaling module in heart that converts extracellular stimuli, in particular mechanical stress and hormonal inputs, into subsequent cellular responses [6], [7]. MAPK signaling consists of a kinase cascade with a tripartite build-up that typically culminates in dual phosphorylation and activation of p38, c-Jun N-terminal kinases and extracellular-signal-regulated kinases (ERKs) [8]. Loss- and gain-of-function experiments in mice addressing single components of the MAPK signaling cascade revealed distinct phenotypes in heart. Enhanced signaling through ERKs led to concentric cardiac hypertrophy with maintained cardiac function [9], [10]. Increased p38 and JNK activity in heart, obtained by cardiomyocyte-specific constitutive expression of their respective upstream kinases, resulted in cardiac fibrosis and ventricular dilation in early adulthood [11]–[13]. In contrast, loss of JNK and p38 signaling led to increased cardiac growth at baseline, as well as in response to pressure-overload [14], [15]. Another recent study has demonstrated that specific loss of JNK1 promoted cardiomyocyte apoptosis and transient cardiac deterioration in the early response to pressure overload [16]. Interestingly, heart-restricted deletion of p38α in mice caused proliferation of adult mammalian cardiomyocytes [17].

MAPK signaling converges into early immediate activation of several transcription factors including myocyte enhancer factor 2 (MEF2) as well as Activator Protein-1 (AP-1). The role of MEF2 in cardiac development and stress adaptation has been extensively explored [18]. In contrast, the role of AP-1 in these processes remains largely unknown. AP-1 comprises a homo or heterodimeric complex that is composed of basic leucine zipper (bZIP) proteins that are subdivided into families of the Jun (c-jun, junB and junD), Fos (c-fos, fosB, fra-1 and fra-2) and the activating transcription factor ATF (ATFa, ATF2, LRF1/ATF3, ATF4 and B-ATF) [19]. Early-immediate up-regulation of AP-1 in response to cardiac hypertrophic stimuli has been reported already in nineties [20]–[23]. But only recently, first evidence for a requirement of AP-1 in the adult heart *in vivo* has been provided using mice that lack and/or ectopically express junD and fra-1, respectively [24], [25]. Preceding *in vitro* studies have mainly focused on the role of c-jun and c-fos in cardiomyocyte growth, two principal factors that are activated by JNK and ERK, respectively. In several of these studies, c-jun and c-fos have been suggested to be required for induction of fetal gene expression and cardiomyocyte hypertrophy in response to different stimuli [26]–[31]. However, studies confirming a requirement of these two transcription factors in these processes *in vivo* are still lacking.

We now provide genetic evidence *in vivo*, employing striated muscle-restricted deletion of *Jun* and *Fos* in mice, that both transcription factors are not essential for postnatal cardiac hypertrophy as well as heart growth in response to mechanical pressure overload. Remarkably however, we found that deletion of *Jun* but not of *Fos* resulted in progressive myocardial fibrosis, cardiomyocyte apoptosis and changes in sarcomeric organization. These alterations were exacerbated in response to mechanical pressure overload resulting in premature heart failure. Conclusively, while c-fos appears to be redundant in heart function, c-jun specifically counteracts pathologic remodeling of the heart subjected to pressure overload.

## Materials and Methods

### Ethics statement

All procedures involving animals were approved by the “Veterinäramt des Kantons Zürich” (approval number 150/2006), and conform to the relevant regulatory standards.

### Generation of *Jun^Δmu^* and *Fos^Δmu^* mice

Floxed *Jun* and *Fos* mice *(Jun^f/f^* and *Fos^f/f^* mice) have been generated by the laboratory of Prof Erwin F. Wagner as previously described [32], [33]. Mice harboring deletion of *Jun* or *Fos* in striated muscle (*Jun^Δmu^* or *Fos^Δmu^*) were obtained by crossing *Jun^f/f^* or *Fos^f/f^* mice with mice carrying Cre recombinase under the control of the creatine kinase promoter (MCK-Cre) [34]. Resulting experimental mice were in a mixed 129/Ola-C57BL/6 background and littermates were used for experiments.

### Mouse experiments

Age- (8- to 12-week-old) and weight- (22–26 g) matched mice in a mixed 129/Ola-C57BL/6 background were subjected to transverse-aortic constriction (TAC) through constriction of the descending aorta using a 27 Gauge needle as previously described [35]. Mice were anesthetized with ketamin/xylazine (2 mg/20 g and 0.2 mg/20 g, respectively) i.p. and analgized with Temgesic (0.003 mg/20 g) i.m. Mice were monitored for 6 weeks after surgery, sacrificed and heart and body weights were determined. Heart functions were assessed by echocardiography 5.5 weeks after surgery. The numbers of experimental animals are indicated in respective figure legends. Reproducibility of the induction of pressure overload by TAC performed by experimenters was previously verified [36].

### Southern blotting and PCR

Genomic DNA was extracted from heart, skeletal muscle and liver of *Fos^f/f^* and *Fos^Δmu^* mice, and from heart, skeletal muscle and kidney of *Jun^f/f^* and *Jun^Δmu^* mice, according to a standard protocol. 20 μg of genomic DNA was digested with XbaI yielding a 6.9 kb fragment for the floxed *Jun* allele and a 3.3 kb fragment for the deleted *Jun* allele. For detection of the bands, a 0.6 kb BamHI fragment from the *Jun* promoter region was used as a probe [37]. 20 μg of genomic DNA was digested with HindIII yielding a 6.3 kb fragment for the floxed *Fos* allele and a 2.9 kb fragment for the deleted *Fos* allele. For detection of the bands, a 0.8 kb BamHI/XbaI EGFP fragment was used as a probe. PCR analysis of genomic DNA isolated from various organs of *Jun^+/+^*, *Jun^f/f^* and *Jun^Δmu^* yielded a 297 bp band corresponding to the wild type alleles, a 344 bp band for the floxed alleles and a 450 bp for the deleted alleles. PCR analysis of genomic DNA isolated from various organs of *Fos^+/+^*, *Fos^f/f^* and *Fos^Δmu^* yielded a 333 bp band corresponding to the wild type alleles, a 433 bp band for the floxed alleles and a 1042 bp for the deleted alleles.

### Echocardiography

Echocardiography was performed as previously described [38]. Briefly, echocardiographic measurements of mice were carried out using an ATL HDI 5000 ultrasound device (Philips Medical Systems) equipped with a 12 MHz phase array linear transducer (L-12-5). M-mode images were used for measurements of IVSd, IVSs, LVPWd, LVPWs, LVIDd, and LVIDs. Fractional shortening (FS) and Ejection Fraction (EF) were calculated using the formulas: FS (%)  =  [(LVIDd – LVIDs)/LVIDd] ×100; EF (%)  =  [(LV Vold – LV Vols)/LV Vold] ×100.

### Mouse neonatal cardiomyocyte culture

Mouse neonatal hearts were isolated as previously described [24]. Briefly, cardiac ventricles were fragmented, digested with ADS buffer containing collagenase (Worthington Biochemical Corp.) and pancreatin (Sigma), and plated in plating medium containing 65% DMEM (Animed), 17% M199 (Animed), 10% Horse Serum (Invitrogen), 5% Fetal Calf Serum (Invitrogen), 2% L-Glutamine (Animed), 1% PS (Invitrogen). After 24 h, medium was changed to maintenance medium (86% DMEM, 10% M199, 1% Horse Serum, 2% L-Glutamine, 1% PS).

### Histology and TUNEL Assay

Hearts were fixed in a 4% formalin solution (Medite) for 24 h at +4°C, embedded in paraffin, and cross-sections of 5 µm thickness were prepared. Sections were stained with H&E or Elastin van Gieson using standard protocols. Sections were also used for TUNEL assays (terminal deoxynucleotide transferase-mediated dUTP nick end labeling) (Roche) to assess the number of apoptotic cells. The staining was performed according to the manufacturer's instructions. The number of apoptotic cells on sections was related to the total heart area on the respective slides. Three independent sections per mouse were analyzed.

### Immunofluorescence and immunochistochemistry

Immunofluorescence and immunohistochemistry on paraffin sections, as well as immunofluorescence on isolated mouse neonatal cardiomyocytes were performed according to standard protocols. The following antibodies were used: α-c-Jun (BD Transduction Laboratories), α-Collagen IV (Cedarlane), α-Periostin (Abcam), α-Actinin 1 (Sigma), α-Titin (kind gift of Dr. E. Ehler, Randall Institute, King's College London, England), α-β-Catenin (kind gift of Dr. E. Ehler Randall Institute, King's College London, England).

### Western blotting

Western blotting using total heart extracts was performed according to standard procedures. The following antibodies were used: α-c-fos (Santa Cruz), α-c-jun (Santa Cruz), α-GFP (Santa Cruz), α-phospho-Smad2 (Cell Signaling), α-Smad2 (Cell Signaling), α-Periostin (Abcam), α-Myotilin (Santa Cruz), α-Tubulin (Sigma), α-GAPDH (Sigma), α-Actin (Sigma).

### Gelatin zymography

Gelatin zymography of total heart proteins was performed as previously described with minor adaptations [39]. Briefly, samples (100 µg of proteins) were mixed with Laemmli sample loading buffer without β-mercaptoethanol and without boiling were loaded on 10% SDS-polyacrylamide gels containing 2 mg/ml gelatin type A from porcine skin (Sigma). After electrophoresis, gels were washed 2 times for 30 minutes in 2.5% Triton X-100 to allow proteins to renature, and then for 10 minutes in 100 mM Tris-HCl pH 7.4. Gels were then incubated at 37°C overnight in developing buffer (50 mM Tris-HCl, pH 7.5, 10 mM CaCl2, 1 µmol/L ZnCl2). Last, to reveal zones of lysis, gels were stained for 30 minutes with 0.5% Coomassie blue R250 and destained for 4 hours with 40%:10% v:v methanol:acetic acid, and then with 5%:7.5% v:v methanol:acetic acid until the stacking gel became colorless.

### Quantitative RT-PCR

RNA was purified from total hearts using TRIzol Reagent (Invitrogen) according to manufacturer's instructions. 5 µg of RNA was used as a template to synthesize cDNA, using Ready-To-Go You-Prime First-Strand Beads (Amersham). Quantitative RT-PCR reactions were set up as recommended by the manufacturer (Roche) and were run and analyzed using the Roche LightCycler 480 system.

### Affymetrix gene expression profiling

Affimetrix gene expression profiling was performed in collaboration with Functional Genomic Center Zürich. Total RNA was extracted from the hearts of 10 weeks old *Jun^f/f^* (n = 2) and *Jun^Δmu^* (n = 2) mice using TRIzol Reagent (Invitrogen). The quality of the RNA was determined with a NanoDrop ND 1000 (NanoDrop Technologies) and a Bioanalyzer 2100 (Agilent). 1 μg of RNA was reverse-transcribed into double-stranded cDNA with One-Cycle cDNA Synthesis Kit (Affymetrix), purified using a Sample Cleanup Module (Affymetrix) and then *in vitro* transcribed in presence of biotinlabeled nucleotides using IVT Labeling Kit (Affymetrix). Then, biotinylated cRNA was purified and its quality and quantity was determined, as described above. 5 μg of biotin-labeled cRNA was fragmented randomly to 35–200 bp and hybridized to GeneChip® GeneChip Mouse Genome 430 2.0 Arrays. The fluorescent intensity emitted by the labeled target was measure in Affymetrix GeneChip Scanner 3000 (Affymetrix). Raw data processing was performed using the Affymetrix AGCC software. Genes with significant expression difference between 2 knock-out mice and 2 wt were selected, based on the average knock-out versus wild-type value greater than 1.3 fold change with a p-value cutoff of 0.05 (using Student's t-test) or differential expression of selected genes which were confirmed by quantitative RT-PCR. The full data set is available in Gene Expression Omnibus data repository, accession number: GSE47898.

### Statistical analysis

Averaged data are presented as means ±SEM. Statistical significance was calculated using an ANOVA with post-hoc Tukey's test and Student unpaired *t* test. Significance was accepted at the level of p<0.05.

## Results

### Eccentric cardiac hypertrophy upon pressure overload in mice lacking *c-Jun* in cardiomyocytes

**Figure 1:**
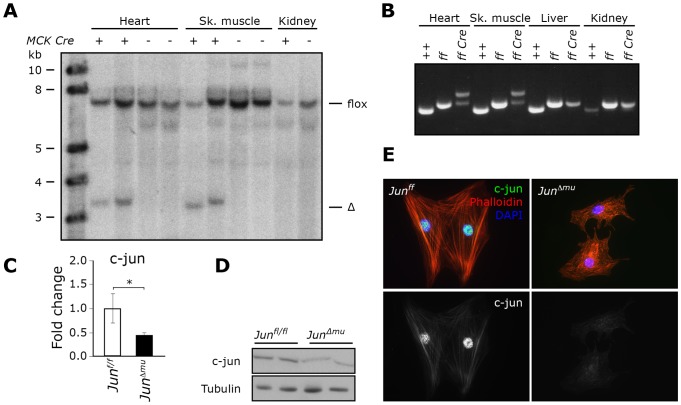
Generation of *Jun^Δmu^* mice. (a) Southern blot analysis of genomic DNA from total heart, skeletal muscle and kidney extracts. Deleted band (Δ band) occurs only in samples from MCK-cre positive heart and skeletal muscle. (b) PCR analysis of genomic DNA. PCR in samples from *Jun^+/+^* (+/+), *Jun^f/f^* (f/f) and *Jun^Δmu^* (f/f Cre) mice yielded a 297 bp band corresponding to the wild type allele, a 344 bp band for the floxed allele and a 450 bp for the Δ allele. (c) Quantitative RT-PCR. *Jun* mRNA levels are down-regulated in total heart extracts from *Jun^Δmu^* mice. (d) Western blot analysis of c-Jun protein levels in total heart extracts of indicated genotypes. (e) Immunofluorescence of isolated mouse neonatal cardiomycoytes. Nuclear localization of c-Jun can be observed in plated neonatal *Jun^f/f^* cardiomyocytes, but not *Jun^Δmu^* cardiomyocytes.

Based on *in vitro* studies, the AP-1 transcription factor c-jun has been suggested to be important in growth and function of the adult heart [40]. To study its role in cardiomyocytes *in vivo*, we generated striated muscle-restricted conditional *Jun* knockout mice using mice carrying floxed alleles of *Jun* that have been crossed to Muscle Creatine Kinase (MCK)-Cre transgenic mice [33], [34]. Southern blotting and PCR confirmed the appearance of deleted alleles in hearts and skeletal muscles of floxed *Jun* mice that were *Cre*-positive but not in non-muscle tissues. *Cre*-negative mice did not reveal any deletion in hearts and skeletal muscles (Figure 1A and B). An floxed band (flox) was present in all samples as recombination was not complete due to the fact that only 30 to 40% of cells in heart and skeletal muscle are muscle cells. Quantitative RT-PCR and Western Blotting, respectively, verified attenuated levels of *Jun* mRNA and reduced abundance of c-jun protein in hearts of *Cre*-positive floxed mice compared to *Cre*-negative floxed control mice (Figure 1C and D). Furthermore, nuclear localization of c-jun protein was detected in isolated primary neonatal cardiomyocytes of floxed control mice (here and after referred to as *Jun^f/f^* mice) but not of conditional *Jun* knockout mice (here and after referred to as *Jun^Δmu^* mice) confirming efficient deletion of *Jun* in cardiomyocytes *in vivo* (Figure 1E).

*Jun^Δmu^* mice were born at Mendelian frequency and presented with normal general health, viability, fecundity, body composition and body weight as compared to control mice (Table S1). To test if in the absence of c-jun in cardiomyocytes, hearts of these animals presents increased susceptibility to the pressure induced heart failure, we subjected mice to mild transaortic constriction (TAC), a widely used model to induce pressure overload and cardiac growth [41].

**Figure 2:**
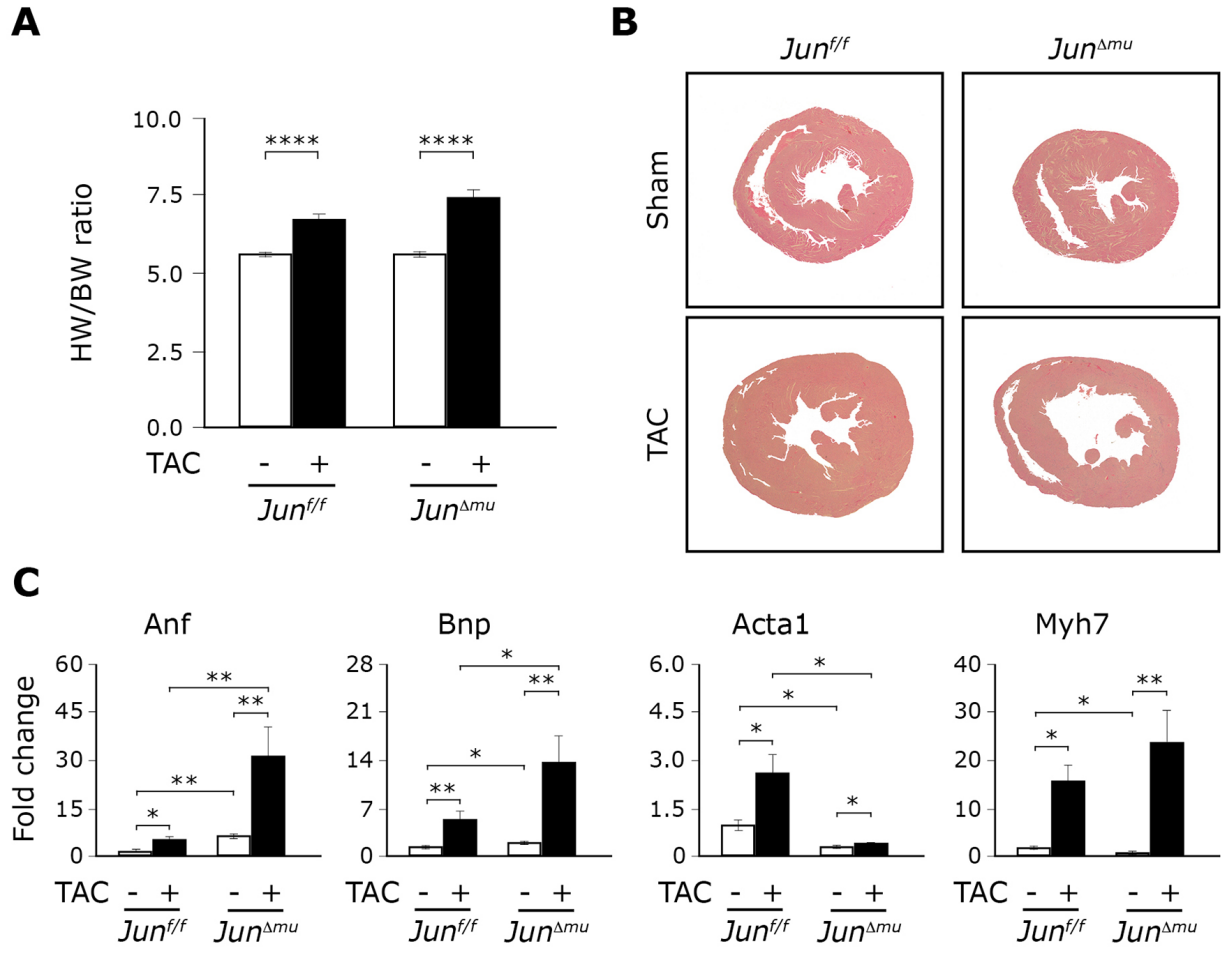
Eccentric cardiac hypertrophy in *cJun^Δmu^* mice upon TAC. (a) H/BW ratios increase of *Jun^Δmu^* and *Jun^f/f^* mice before and after TAC. Data are presented as values ± SEM. (****) p<0.0001; n = 11–12 per group. (b) Histological analyses. H&E staining shows TAC-induced concentric growth of the heart in *Jun^f/f^* mice and the heart dilation in *Jun^Δmu^* mice. (c) Relative expression of indicated hypertrophic markers in hearts isolated from sham or TAC operated *Jun^f/f^* and *Jun^Δmu^* mice assessed by quantitative RT-PCR. Data are presented as values ± SEM. (*) p<0.05, (**) p<0.01; n = 5 per group.

Heart to body weight (H/BW) ratios significantly and similarly increased in *Jun^Δmu^* and *Jun^f/f^* mice upon TAC compared to sham-operated littermates (Figure 2A). H/BW ratios correlated with the average cross sectional area of cardiomyocytes *in situ* confirming a similar growth response in heart upon TAC (Figure S1 A and B). However, histological cross-sections revealed that hearts of *Jun^Δmu^* mice were markedly dilated 6 weeks after TAC compared to hearts of *Jun^f/f^* that presented with concentric myocardial growth (Figure 2B). No differences between hearts cross sections of sham-operated mice of both genotypes were detected. These observations could be confirmed by echocardiography (Table 1). Indeed, hearts of both genotypes showed significant muscle growth in response to TAC as an increase in left ventricular posterior wall (LVPW) thicknesses was observed. In comparison to sham-operated mice, left ventricular internal dimensions (LVID) were significantly decreased upon TAC in *Jun^f/f^* mice. In contrast, they were markedly increased in *Jun^Δmu^* mice indicating concentric and eccentric hypertrophy, respectively. As a consequence, cardiac performance was impaired in TAC-operated *Jun^Δmu^* mice as significant decreases in fractional shortening (FS) and ejection fraction (EF) were observed compared to sham-operated *Jun^Δmu^* mice and TAC-operated *Jun^f/f^* mice. No differences in echocardiographic parameters were detected between sham-operated mice of both genotypes.

**Table 1:** Echocardiographic analyses in *Jun^Δmu^* mice after TAC.

	*Jun^f/f^*		*Jun* ^Δ*mu*^	
Data measure	Sham	TAC	Sham	TAC
HR, bpm	435±23	467±22	450±25	490±39
LVPWd, mm	0.70±0.002	0.86±0.057 #	0.71±0.021	0.85±0.045 †
LVPWs, mm	0.93±0.009	1.12±0.032 #	0.96±0.018	1.06±0.028 †
LVIDd, mm	4.20±0.027	3.98±0.077 #	4.13±0.066	4.51±0.253
LVIDs, mm	2.99±0.039	2.69±0.069 #	2.82±0.069	3.56±0.280 †§
FS, %	28.83±0.62	32.42±0.73 #	31.90±0.80	21.46±2.01 †§
EF, %	55.84±0.96	61.29±1.08 #	60.39±1.20	43.53±3.56 †§

All values are shown as mean ± SEM. n = 5–6 per group. p<0.05 is indicated as: # WT TAC vs WT sham; † KO TAC vs KO sham; § KO TAC vs WT TAC. HR, Heart rate; LVPWd, Left ventricular posterior wall in diastole; LVPWs, Left ventricular posterior wall in systole; LVIDd, Left ventricular internal diameter in diastole; LVIDs, Left ventricular internal diameter in systole; FS, Fractional Shortening; EF, Ejection Fraction.

Left ventricular remodeling upon TAC entails induction of hypertrophic marker genes such as for example atrial natriuretic factor (*Anf*), brain natriuretic peptide (*Bnp*), myosin heavy polypeptide 7 (*Myh7*) and skeletal muscle alpha-actin (*Acta1*). Quantitative RT-PCR revealed that mRNAs of *Anf* and *Bnp* were significantly increased in TAC-operated hearts of both *Jun^f/f^* and *Jun^Δmu^* mice (Figure 2C). Importantly, cardiac expression of *Anf* and *Bnp* was significantly greater in *Jun^Δmu^* compared to *Jun^f/f^* mice upon TAC. Interestingly, *Anf* and *Bnp* were also significantly enhanced in sham-operated hearts of *Jun^Δmu^* mice compared to hearts of *Jun^f/f^* mice. In contrast, *Acta1* was expressed at significantly lower levels in hearts of *Jun^Δmu^* mice at baseline, and remained low in hearts of *Jun^Δmu^* mice compared to hearts of *Jun^f/f^* mice in response to pressure-overload (Figure 2C). *Myh7* was expressed at significantly lower levels in native hearts of *Jun^Δmu^* mice, but was significantly and similarly induced in hearts of both *Jun^Δmu^* and *Jun^f/f^* mice upon TAC (Figure 2C). These data suggest that deletion of *Jun* in cardiomyocytes resulted in basal changes in expression of hypertrophic marker genes without obvious morphological signs of cardiac hypertrophy and dysfunction. Expression of these genes remained dramatically altered upon left ventricular pressure-overload and was associated with maladaptive hypertrophy.

### Normal cardiac function in mice lacking *Fos* in cardiomyocytes

The AP-1 transcription factor c-fos has also been extensively discussed as a key player in cardiac function [40]. To study its role in cardiomyocytes *in vivo*, we used *Fos* floxed mice and crossed them to MCK-Cre transgenic mice. Southern blotting, PCR and Western blotting confirmed efficient and specific deletion of *Fos* in striated-muscle cells (Figure S2 A, B and C). The floxed *Fos* allele is generated in the way that its removal leads to GFP expression [33] that was also detected by Western Blotting in hearts of *Cre*-positive floxed mice (Figure S2 C). *Fos^Δmu^* mice were born at Mendelian frequency and presented with normal general health, viability, fecundity, body composition and body weight as compared to *Fos^f/f^* mice (Table S2). We subsequently subjected mice to TAC. H/BW ratios significantly and equally increased in both *Fos^f/f^* and *Fos^Δmu^* mice after TAC compared to sham-operated mice (Figure S3 A). Histological analyses of heart cross sections displayed an apparent increase in heart size after TAC compared to sham-operated mice of both genotypes. Importantly, no ventricular dilation could be seen in pressure-overloaded *Fos^Δmu^* and *Fos^f/f^* mice (Figure S3 B). These findings were in line with our subsequent analyses by echocardiography. Sonographic assessment revealed significant and comparable increases of cardiac wall dimensions upon TAC in *Fos^Δmu^* and *Fos^f/f^* mice. FS and EF were maintained in both genotypes after TAC (Table S3). Quantitative RT-PCR revealed significant and similar increases of the mRNA of hypertrophy marker genes in hearts of both genotypes compared to sham-operated mice. We observed a tendency towards enhanced induction of these genes in response to TAC in *Fos^Δmu^* compared to *Fos^f/f^* mice, which was however only reaching significance for *Myh7* (Figure S3 C). Thus, deletion of *Fos* in cardiomyocytes does not alter cardiac development, postnatal heart growth as well as cardiac hypertrophy in response to mechanical pressure overload.

### Impaired myocardial remodeling in hearts lacking *Jun* in cardiomyocytes

**Figure 3:**
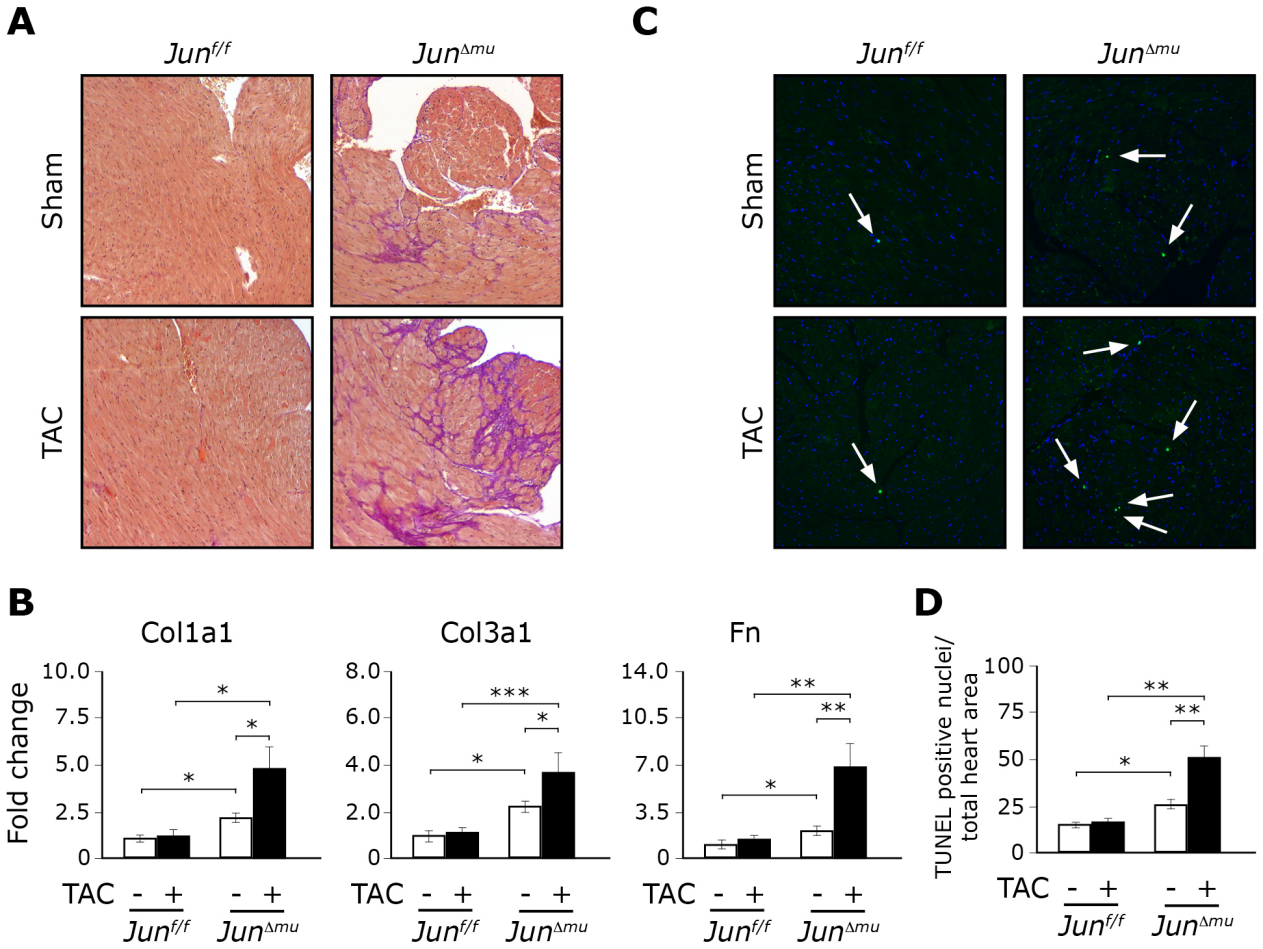
*Jun^Δmu^* mice display impaired myocardial remodeling. (a) Histological analyses. EvG staining reveals mild spontaneous fibrosis in hearts of *Jun^Δmu^* mice, being markedly enhanced after TAC. (b) Relative expression of indicated fibrotic markers assessed by quantitative RT-PCR in hearts isolated from sham or TAC operated *Jun^Δmu^* and *Jun^f/f^* mice. Data are presented as values ± SEM. (*) p<0.05, (**) p<0.01, (***) p<0.001; n = 5 per group. (c) TUNEL staining of heart cross sections. TAC does not induce apoptosis in hearts of *Jun^f/f^* mice. *Jun^Δmu^* mice show slightly more apoptotic cardiomyocytes already at baseline, while the apoptotic rate markedly increased in hearts of *Jun^Δmu^* mice upon TAC. (d) Quantification of TUNEL positive nuclei does in sham or TAC operated animals of indicated genotypes. Data are presented as values ± SEM. (*) p<0.05, (**) p<0.01.

In the following, we addressed cellular mechanisms underlying premature heart failure in *Jun^Δmu^* mice upon mechanical pressure overload. Enhanced myocyte loss and cardiac fibrosis are key factors promoting progression of cardiac hypertrophy to heart failure [42], [43]. An Elastin van Gieson (EvG) stain that allowed for a better differential analysis of nuclei, connective tissue, muscle and elastic fibers revealed widespread myocardial fibrosis in hearts of *Jun^Δmu^* mice, while no foci of apparent collagen deposition were detectable in hearts of *Jun^f/f^* 6 weeks after TAC. Fibrosis in hearts of *Jun^Δmu^* mice was evident already at baseline (in 12 weeks old *Jun^Δmu^* mice) but was markedly aggravated upon TAC (Figure 3A). Interstitial fibrosis was associated with increased amounts of collagen type I (*Col1a1*), collagen type III (*Col3a1*) and fibronectin (*Fn*) (Figure 3B), all of which are commonly recognized as fibrotic markers in heart [44]–[47]. At baseline, *Jun^Δmu^* mice showed significant up-regulation of *Col1a1*, *Col3a1* and *Fn* mRNA levels in native hearts, when compared to *Jun^f/f^* mice (Figure 3B). TAC further enhanced the expression of these genes in hearts of *Jun^Δmu^* mice but not in hearts of *Jun^f/f^* mice confirming interstitial fibrosis. Myocardial fibrosis is frequently accompanied by enhanced cardiomyocyte apoptosis. Indeed, TUNEL staining confirmed significantly increased numbers of apoptotic cardiomyocytes in *Jun^Δmu^* mice upon TAC, when compared to sham-operated mice as well as TAC-operated *Jun^f/f^* mice. Interestingly, already at baseline, *Jun^Δmu^* mice showed significantly more TUNEL positive nuclei in hearts than *Jun^f/f^* mice. The numbers of apoptotic nuclei in the hearts of *Jun^f/f^* mice did not change upon TAC after 6 weeks (Figure 3C and D).

Several matrix metalloproteinases (Mmps) that have been identified within the myocardium are dysregulated in heart failure and transcriptional regulation of Mmps by AP-1 transcription factors has been reported [43]. Cardiac mRNA levels of *Mmp2* and *Mmp14* were significantly upregulated in *Jun^Δmu^* mice upon TAC compared to TAC-operated *Jun^f/f^* and sham-treated mice of both genotypes (Figure S4 A). *Mmp9* mRNA levels were slightly increased in native hearts of *Jun^Δmu^* compared to *Jun^f/f^* mice, however its levels in hearts of TAC operated *Jun^Δmu^* mice were not altered compared to TAC operated *Jun^f/f^* hearts (Figure S4 A). Other cardiac Mmps, Mmp1, Mmp13 and Mmp3 were not found deregulated (data not shown). Gelatin zymography assays confirmed that mRNA expression patterns correlated with activities of Mmp2 and Mmp9 in hearts of both genotypes (Figure S4 B).

Fibrosis and impairment of the heart function is often associated with aging. To test if deletion of *Jun* in heart accelerates aging of this organ, we stained cross sections of hearts isolated from one year old *Jun^Δmu^* mice and *Jun^f/f^* control animals with EvG staining. We observed minimal progression of fibrosis in hearts of *Jun^Δmu^* mice while fibrotic remodeling was absent in hearts of control animals (Figure S5). Consistently, heart function of one year old *Jun^Δmu^* mice was comparable to the aged matched control animals as revealed by echocardiography analyzes (Table S4).

Overall, cardiomyocyte-specific deletion of *Jun* resulted in deleterious myocardial remodeling that involved increased fibrosis associated with enhanced degradation of extracellular matrix protein and programmed cell death leading to premature heart failure under stress conditions. Although our data indicate that deficiency of *Jun* in heart of mice is not sufficient to provoke failure of this organ up to the age of one year, we cannot exclude that further aging would result in the impairment of cardiac function in these animals.

### Upregulation of extracellular matrix and downregulation of cytoskeletal genes in hearts of *Jun^Δmu^* mice

**Figure 4:**
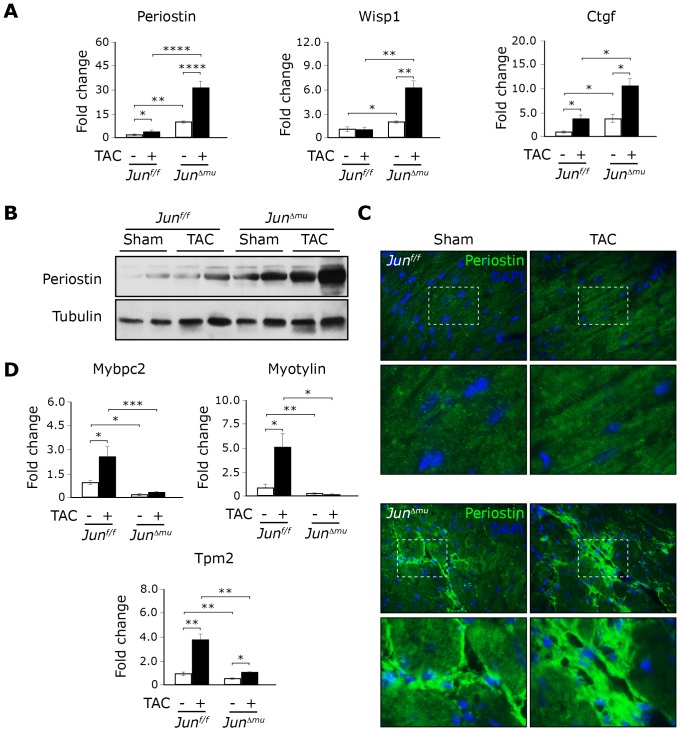
Upregulation of extracellular matrix proteins and downregulation expression of sarcomeric associated proteins in hearts of *Jun^Δmu^* mice. (a) Expression of indicated genes in hearts of sham or TAC operated mice of indicated genotypes. Data are presented as values ± SEM. (*) p<0.05, (**) p<0.01, (***) p<0.001, (****) p<0.0001; n = 5 per group. (b) Western blot analyses of Periostin abundancein hearts from sham and TAC operated *Jun^Δmu^* and *Jun^f/f^* mice. Tubulin was used as a loading control for the extracts. (c) Immunoflorescence staining for Periostin in cross sections of hearts from sham and TAC operated animals of indicated genotypes.

In an attempt to identify specific genes that might be globally affected in the absence of c-jun in cardiomyocytes, we compared gene expression in non-stimulated hearts from *Jun^f/f^* and *Jun^Δmu^* mice using Affymetrix™ oligonucleotide expression arrays. We performed these analyzes in non-stimulated conditions as hearts from *Jun^Δmu^* mice already presented a mild phenotype (increased fibrosis and altered expression hypertrophic markers) but did not present functional defects. We obtained a list of 543 genes, with 211 genes up-regulated and 332 genes down-regulated, for which differential expression was greater or lesser than 1.3 fold in hearts of *Jun^Δmu^* mice when compared to hearts of *Jun^f/f^* mice. Importantly, we analyzed 33 genes by QPCR on the mRNA isolated from hearts of *Jun^f/f^* and *Jun^Δmu^* mice and confirmed deregulation of 18 of them (Table S5). Consistent with marked fibrosis in hearts of *Jun^Δmu^* mice, we found several extracellular matrix genes expression of which was increased compared to hearts of *c-Jun^f/f^* mice. Among other extracellular matrix genes, upregulation of periostin, connective tissue growth factor (Ctgf) and WNT1 inducible signaling pathway protein 1 (Wisp1) were most interesting, since their involvement in triggering myocardial fibrosis and heart failure has been previously established [48]–[52]. Quantitative RT-PCR confirmed that all genes were expressed at significantly higher levels in sham-operated hearts of *Jun^Δmu^* mice than in hearts of *Jun^f/f^* mice (Figure 4 A–C). Although periostin, Ctgf and Wisp1 expression was significantly enhanced upon TAC in hearts of both *Jun^f/f^* and *Jun^Δmu^* mice when compared to sham-operated mice, their expression in TAC-operated hearts of *Jun^Δmu^* mice was markedly higher than in hearts of *Jun^f/f^* mice subjected to the same procedure. Enhanced periostin expression in sham- and TAC-operated *Jun^Δmu^* mice could also be confirmed at the protein level by Western blotting (Figure 4 B). Moreover, immunofluorescence revealed marked deposition of periostin in the interstitium of the myocardium of *Jun^Δmu^* mice, while its accumulation was not detectable in hearts of *Jun^f/f^* mice (Figure 4 C).

Most remarkably, 38 of deregulated candidates in hearts from *Jun^Δmu^* mice were associated with muscle contraction and cytoskeleton organization. Among them myosin binding protein C 2 (Mybpc2), myotilin and β-tropomyosin 2 draw our attention as they are accessory components of the sarcomere [53]–[55]. Sarcomeres are the principle components of the cardiac contractile machinery composed of thick and thin filaments [56]. Importantly, sarcomeric organization increases upon hypertrophic stimuli and its disorganization is often associated with advanced cardiac hypertrophy and heart failure [57]. We studied in details the expression of Mybpc2, myotylin and β-tropomyosin 2 by QPCR in hearts from control *Jun^f/f^* and *Jun^Δmu^* mice. Expression of all 3 genes was markedly downregulated in unstimulated hearts from *Jun^Δmu^* mice compared to unstimulated hearts from *Jun^f/f^*. Moreover, their expression was markedly induced in hearts from *Jun^f/f^* mice subjected to TAC but not in *Jun^Δmu^* mice on which the same intervention was performed (Figure 4 D).

Thus, loss of c-Jun in cardiomyocytes leads to deregulation of several genes that are required for organization of the sarcomere. c-Jun is also required for their induction upon TAC.

### c-jun regulates sarcomere organization

**Figure 5:**
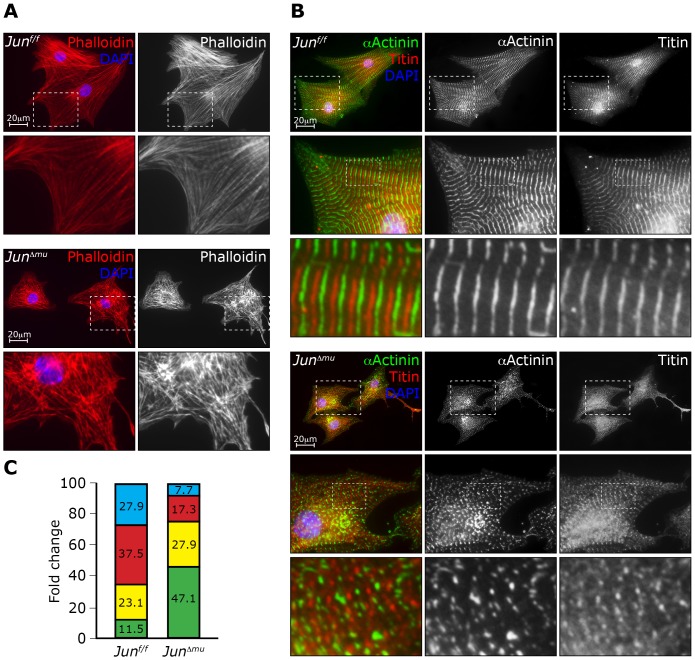
Altered sarcomeric structure of isolated neonatal *Jun^Δmu^* cardiomyocytes. (a) Immunofluorescent staining. Phalloidin staining shows defects in structural organization of *Jun^Δmu^* cardiomyocytes as compared to *Jun^f/f^* cardiomyocytes. (b) Immunofluorescent staining. alpha actinin (αActinin) and titin staining show disruption of Z-line and M-band struture in *Jun^Δmu^* cardiomyocytes as compared to *Jun^f/f^* cardiomyocytes. (c) Relative percent of cardimyocytes presenting different cytoskeleton organization: blue bars – fully organize cytoskeleton, red – well organized, yellow – moderately and green – poorly organized cytoskeleton.

In our microarray expression analysis several genes that encode for components of the thick and thin filaments were found to be deregulated in hearts in the absence of *Jun* in cardiomyocytes. We therefore hypothesized that deletion of c-jun in cardiomyocytes affects sarcomere organization. The cultured cardiomyocyte system is particularly suited to assess sarcomere organization since morphological changes can be easily recognized since it provides much higher resolution compared to the *in vivo* system [57]. We therefore isolated neonatal cardiomyocytes from *Jun^Δmu^* and *Jun^f/f^* mice and performed immunofluorescent stainings using phalloidin that binds F-actin, as well as antibodies against α-actinin 1 that localizes to the Z-disc and against M-band titin. Phalloidin staining revealed a marked disarray of polymerized actin fibers and the sarcomeric structure appeared rudimentary in cardiomyocytes isolated from neonatal *Jun^Δmu^* mice. In contrast, actin fibers were assembled in a more organized fashion into sarcomeres in control cardiomyocytes (Figure 5A). Sarcomeric α-actinin 1 and M-band titin stainings displayed a punctuated, thin and rudimentary sarcomeric structure in cardiomyocytes lacking *Jun* (Figure 5B), while control cells showed well-organized sarcomeres. To better describe the observed phenotype, we defined four categories of cytoskeleton organization (Figure 5C). 47% of c-jun-deficient cardiomyocytes presented poorly organized cytoskeleton compared to 11.5% of control cells. Conversely, 27.9% and 37.5% of control cardiomyocytes presented fully or well organized cytoskeleton while only 7.7% and 17.3% of c-Jun deficient cells fell into these 2 categories, respectively. Similar numbers of c-jun-deficient and control cardiomyocytes (27.9 and 23.1% respectively) presented moderately organized cytoskeleton. Overall, the quantification revealed decreased number of cells presenting organized and increased disorganized cytoskeleton in the absence of c-jun (Figure 5C).

These results thus support a requirement of c-jun in cytoskeletal remodeling in cardiomyocytes and provide evidence that changes in expression profile of genes involved in cytoskeleton organization in c-jun-deficient cardiomyocytes result in functional changes observed in mice.

## Discussion

Our data demonstrate that c-Jun is necessary for maintenance of proper organization of cytoskeleton and sarcomeric structure in cardiomyocytes and protects the heart from pathologic remodeling and thus from heart dilation and heart failure, in particular under stress conditions.

c-jun and c-fos are members of the AP-1 transcription factor family. Both transcription factors have been previously shown to be upregulated upon mechanical and pharmacological hypertrophic stimuli [23], [24], [58]. However, functional relevance for this regulation has not been evidenced thus far. Our results indicate that c-jun is required for maintaining heart function during a hypertrophic response, while c-fos is dispensable. The fact that we did not observe any functional alteration in mice lacking c-fos in hearts during hypertrophic response might be explained by rapid upregulation of another member of the AP-1 family, fra-1 upon hypertrophic stimuli [24]. In fact, a previous study demonstrated that fra-1 can rescue bone development defects of c-fos-deficient mice indicating that the function of these 2 proteins might be partially redundant [59]. Moreover, fra-1 deficiency in heart does not alter the hypertrophic response [24], further supporting the possibility that functions of c-fos and fra-1 might be redundant in heart.

Previous studies implicated c-jun N-terminal kinase (JNK) in maintaining heart function after mechanical pressure overload [16], [60]. JNK activates c-jun transcriptional activity by promoting its phosphorylation [8]. Interestingly, deletion of JNK1 or the upstream kinase Mitogen-activated protein kinase kinase kinase (MEKK1) resulted in a maladaptive response to TAC. Specifically, in the absence of JNK1 or MEKK1 hearts subjected to TAC prematurely decompensated due to an increase in cardiomyocytes apoptosis. We obtained similar results in hearts of *Jun^Δmu^* mice subjected to TAC as compared to control hearts. Therefore, our results indicate that the MEKK1–JNK1 signaling cascade might promote heart function upon TAC, at least partially, by activating c-jun.

In an attempt to globally identify targets of c-jun in heart, we have also performed expression arrays. Among other putative targets of c-jun, we identify extracellular matrix proteins periostin, Wisp1 and Ctgf being increased in the absence of c-jun in hearts. Expression of these 3 genes was further enhanced in c-Jun deficient hearts subjected to TAC. Importantly, expression of these genes has been previously implicated in the regulation of heart function [48]–[52]. In particular periostin has been broadly discussed in the context of heart function. Whether it promotes cardiomyocytes proliferation and cardiac healing after infarction remains the matter of debate [48], [52], [61]. Importantly, however, periostin was shown to promote heart dilation [51]. Therefore the increase of periostin expression and protein levels in c-jun-deficient hearts might contribute to heart failure. Unfortunately, with present data, we cannot answer if c-Jun directly promotes periostin, Wisp1 and Ctgf expression, or whether increased expression of these three genes was an indirect consequence of heart dysfunction caused by loss of c-jun in cardiomyocytes.

Expression arrays revealed that genes involved in regulation of sarcomere organization constitute a group of other potential c-jun transcriptional targets. Particularly, expression of Mybpc2, myotilin and β-tropomyosin 2 were found downregulated in the hearts of *Jun^Δmu^* mice compared to hearts of *Jun^f/f^* mice. Moreover, expression of these genes in *Jun^Δmu^* hearts was not increased upon TAC like in hearts from control animals. Likewise, based on our results we cannot determine whether c-jun regulates these genes in a direct or indirect fashion. However, the c-jun-dependent control of genes involved in regulation of sarcomere organization prompted us to investigate the cytoskeleton and sarcomere structure functionally. Remarkably, c-jun-deficient cardiomyocytes presented disarrangement of sarcomeres and cytoskeleton. Disarrangement of sarcomeres is a hallmark of advanced heart hypertrophy and heart failure, while rapid hypertrophic stimuli increase cytoskeleton and sarcomeres organization [57]. Here, we thus identified a new transcription factor required for proper expression of components of the sarcomeric machinery and therefore for sarcomere function.

In addition to the identification of new cellular functions of c-jun in regulation of sarcomeres organization, our work revealed that deletion of c-jun in cardiomyocytes is associated with increased rate of apoptosis and fibrosis in heart. Depending on the cell type, c-jun has been reported to prevent or to promote apoptosis. In neuronal cells c-jun is required for induction of apoptosis. In contrast, c-jun is necessary for survival of hepatoblasts, hepatic tumor cells and its deletion potentiates UV and TNFα induced apoptosis of mouse embryonic fibroblasts [19], [37], [62]–[64]. Major mechanism of c-jun mediated suppression of apoptosis is by attenuating expression of pro-apoptotic protein p53 and its target gene *noxa* [37]. Interestingly, recent studies point towards p53 as a central molecule in mediating cardiomyocyte apoptosis and heart failure (for review see [65]). Although in our study deletion of *Jun* in heart was restricted to cardiomyocytes, based on our results we cannot define whether deletion of *Jun* promotes apoptosis of cardiomyocytes directly or rather cardiomyocytes deficient for this protein secrete factor/-s which promotes apoptosis of the neighboring cells in heart. Defined role of c-jun in suppressing apoptosis in other cell types in cell autonomous manner would implicate that c-jun might suppress apoptosis of cardiomyocytes directly. Based on our results we cannot point out the potential pro-apoptotic factor secreted by *Jun* deficient cardiomyocytes. Secreted proteins identified in our study as being upregulated in the absence of c-jun in cardiomyocytes (Periostin, Wisp1 and Ctgf) had been implicated previously as a factors rather protecting cells from apoptosis [66]–[71] and their increased expression in the absence of c-jun might represent mechanism to counteract apoptosis of the heart cells. Similarly, in our system we cannot define whether fibrosis observed in the absence of c-jun in cardiomyocytes is a consequence of increased apoptosis of these cells or rather increased secretion of fibrogenic mediators by c-jun deficient cardiomyocytes.

In conclusion, our study led to the identification of c-jun as a new transcription factor preventing cytoskeleton dysfunction, loss of cardiomyocytes and cardiac fibrosis, which constitute hallmarks of maladaptive cardiac growth leading to heart dilation and failure.

## Supporting Information

Figure S1 **Quantification of cardiomyocyte cross-sectional area.** (A) Immunochistochemical staining of heart cross-sections for collagen IV. (B) Quantification of cardiomyocyte cross-sectional area (CSA). Cardiomyocytes from *Jun^f/f^* and *Jun^Δmu^* mice showed a similar increase in cross-sectional area (CSA) after TAC. Data are presented as values ± SEM. (**) p<0.01; 5 mice per group were analyzed 1000 cardiomyocytes per mouse were quantified.
(TIF)

Figure S2 **Generation of** ***Fos^Δmu^*** **mice.** (A) Southern blot analysis of genomic DNA from total heart, skeletal muscle and liver extracts. Deleted band (Δ) occurs only in MCK-cre positive samples from heart and skeletal muscle, while floxed band (flox) is present in all samples. (B) PCR analysis of genomic DNA. PCR in samples from *Fos^+/+^* (+/+), *Fos^f/f^* (f/f) and *Fos^Δmu^* (f/f Cre) mice yielded a 333 bp band corresponding to the wild type alleles, a 433 bp band for the floxed alleles and a 1042 bp for the deleted alleles. (C) Western blot analysis of c-Fos protein levels in total heart extracts. Significant decrease of c-Fos is seen in hearts from *Fos^Δmu^* mice as compared to *Fos^f/f^* mice. Expression of Cre-recombinase in the heart leads to expression of GFP. Actin was used as a loading control.
(TIF)

Figure S3 ***Fos^Δmu^*** **mice show concentric heart hypertrophy upon TAC.** (A) H/BW ratio increases significantly in both *Fos^f/f^* and *cFos^Δmu^* mice upon TAC. Data are presented as values ± SEM. (*) p<0.05; n = 4–6 per group. (B) Histological analyses. H&E staining of heart cross-sections shows slight increase in left-ventricle wall thickness in both TAC-operated groups. (C) Relative expression of hypertrophic markers assessed by quantitative RT-PCR. *Anf, Bnp*, *Acta1*, and *Myh7* are re-expressed in hypertrophied hearts of *Fos^f/f^* and *Fos^Δmu^* mice. (*) p<0.05, (**) p<0.01, (***) p<0.001, (****) p<0.0001; n = 4–6 per group.
(TIF)

Figure S4 **TAC-induced cardiac fibrosis in** ***Jun^Δmu^*** **mice is associated with enhanced MMPs expression and activity.** (A) Relative expression of myocardial MMPs (as indicated) assessed by quantitative RT-PCR, in sham and TAC operated mice from indicated genotypes. Data are presented as values ± SEM. (*) p<0.05, (**) p<0.01; n = 5 per group. (B) Gelatin zymography on total heart protein extracts. TAC-operated *Jun^Δmu^* mice show greatly increased activity of MMP-2 (arrow at 66 kDa), and slightly decreased activity of MMP-9 (arrow at 92 kDa) in hearts as compared to sham-operated mice and TAC-operated *Jun^f/f^* mice. No difference in MMP-2 and MMP-9 activity in hearts is observed between TAC operated *Jun^f/f^* mice, when compared to sham-operated controls, as well as sham-operated *Jun^f/f^* and *^JunΔmu^* mice.
(TIF)

Figure S5 **Hearts of one year old** ***Jun^Δmu^*** **mice present spontaneous fibrosis.** (A) Normal H/BW ratio of one year old *Jun^Δmu^* mice. (B) Histological analyses. EvG staining reveals spontaneous fibrosis in hearts of one year old *Jun^Δmu^* mice.
(TIF)

Table S1 **Body and organs weights in adult** ***Jun*** ^Δ***mu***^ **mice and corresponding control mice.**
(DOC)

Table S2 **Body and organs weights in adult** ***Fos*** ^Δ***mu***^ **mice and corresponding control mice.**
(DOC)

Table S3 **Echocardiographic analyses in** ***Fos*** ^Δ***mu***^ **mice after TAC.**
(DOC)

Table S4 **Echocardiographic assessment of heart function in one year old** ***Jun*** ^Δ***mu***^ **and corresponding control mice.**
(DOC)

Table S5 (XLSX)
