# Supplementary figures and images for: The AP-1 Transcription Factor c-Jun Prevents Stress-Imposed Maladaptive Remodeling of the Heart
Source: PLoS One. 2013 Sep 10;8(9):e73294. doi: 10.1371/journal.pone.0073294 (PMC3769267; doi:10.1371/journal.pone.0073294)

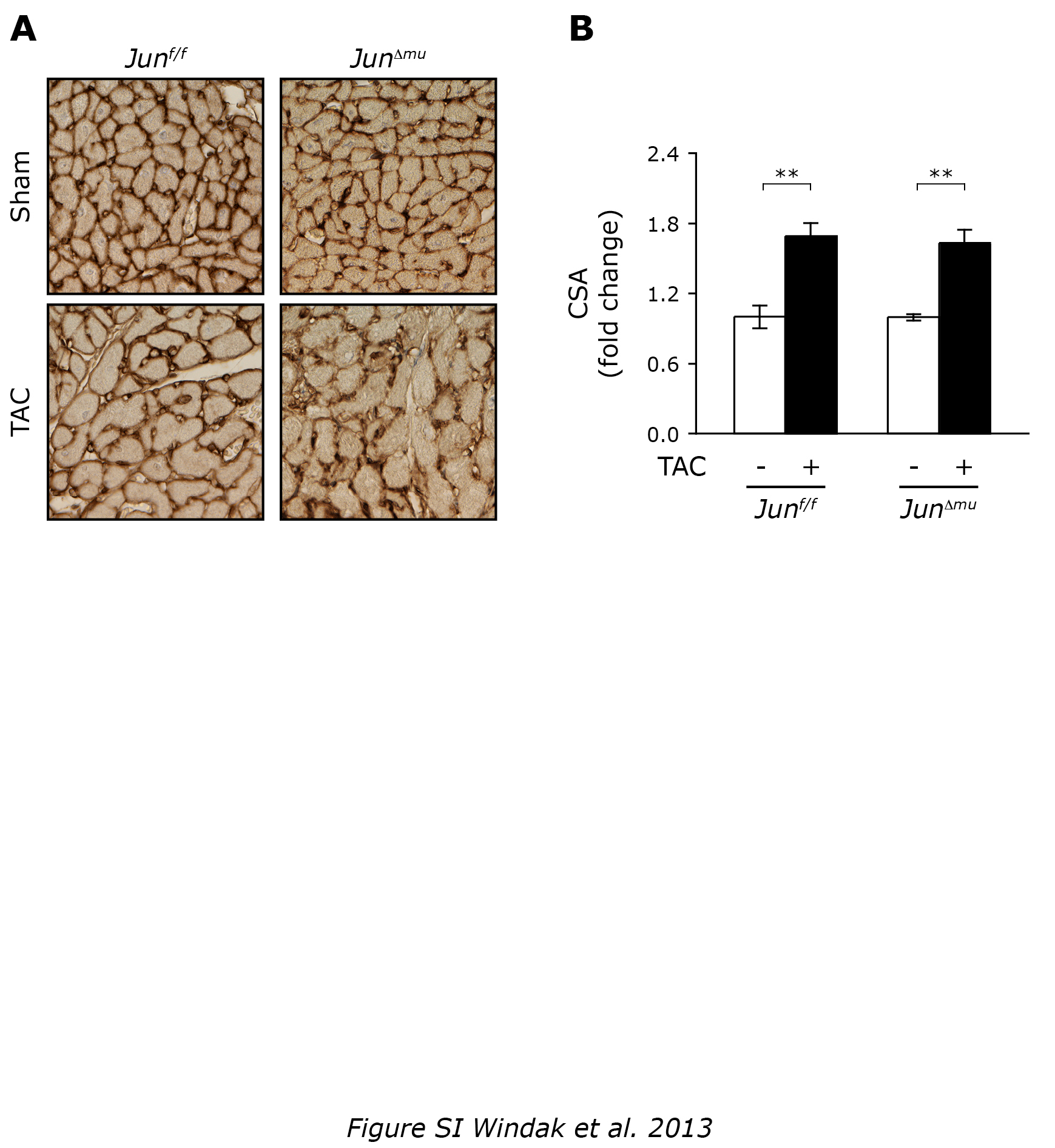

Supplement: Figure S1 — Quantification of cardiomyocyte cross-sectional area. (A) Immunochistochemical staining of heart cross-sections for collagen IV. (B) Quantification of cardiomyocyte cross-sectional area (CSA). Cardiomyocytes from Junf/f and JunΔmu mice showed a similar increase in cross-sectional area (CSA) after TAC. Data are presented as values ± SEM. (**) p<0.01; 5 mice per group were analyzed 1000 cardiomyocytes per mouse were quantified. (TIF) [file pone.0073294.s001.tif]

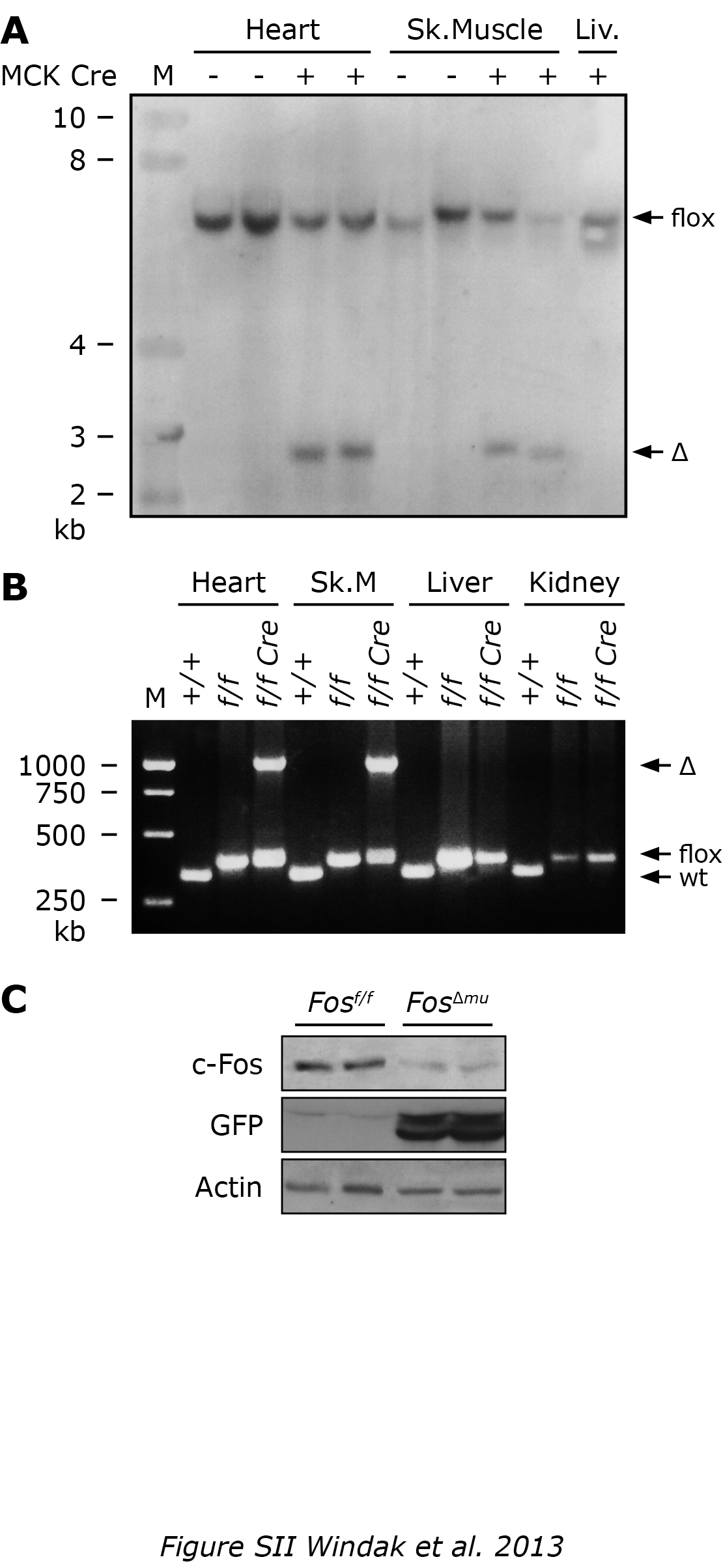

Supplement: Figure S2 — Generation of FosΔmu mice. (A) Southern blot analysis of genomic DNA from total heart, skeletal muscle and liver extracts. Deleted band (Δ) occurs only in MCK-cre positive samples from heart and skeletal muscle, while floxed band (flox) is present in all samples. (B) PCR analysis of genomic DNA. PCR in samples from Fos+/+ (+/+), Fosf/f (f/f) and FosΔmu (f/f Cre) mice yielded a 333 bp band corresponding to the wild type alleles, a 433 bp band for the floxed alleles and a 1042 bp for the deleted alleles. (C) Western blot analysis of c-Fos protein levels in total heart extracts. Significant decrease of c-Fos is seen in hearts from FosΔmu mice as compared to Fosf/f mice. Expression of Cre-recombinase in the heart leads to expression of GFP. Actin was used as a loading control. (TIF) [file pone.0073294.s002.tif]

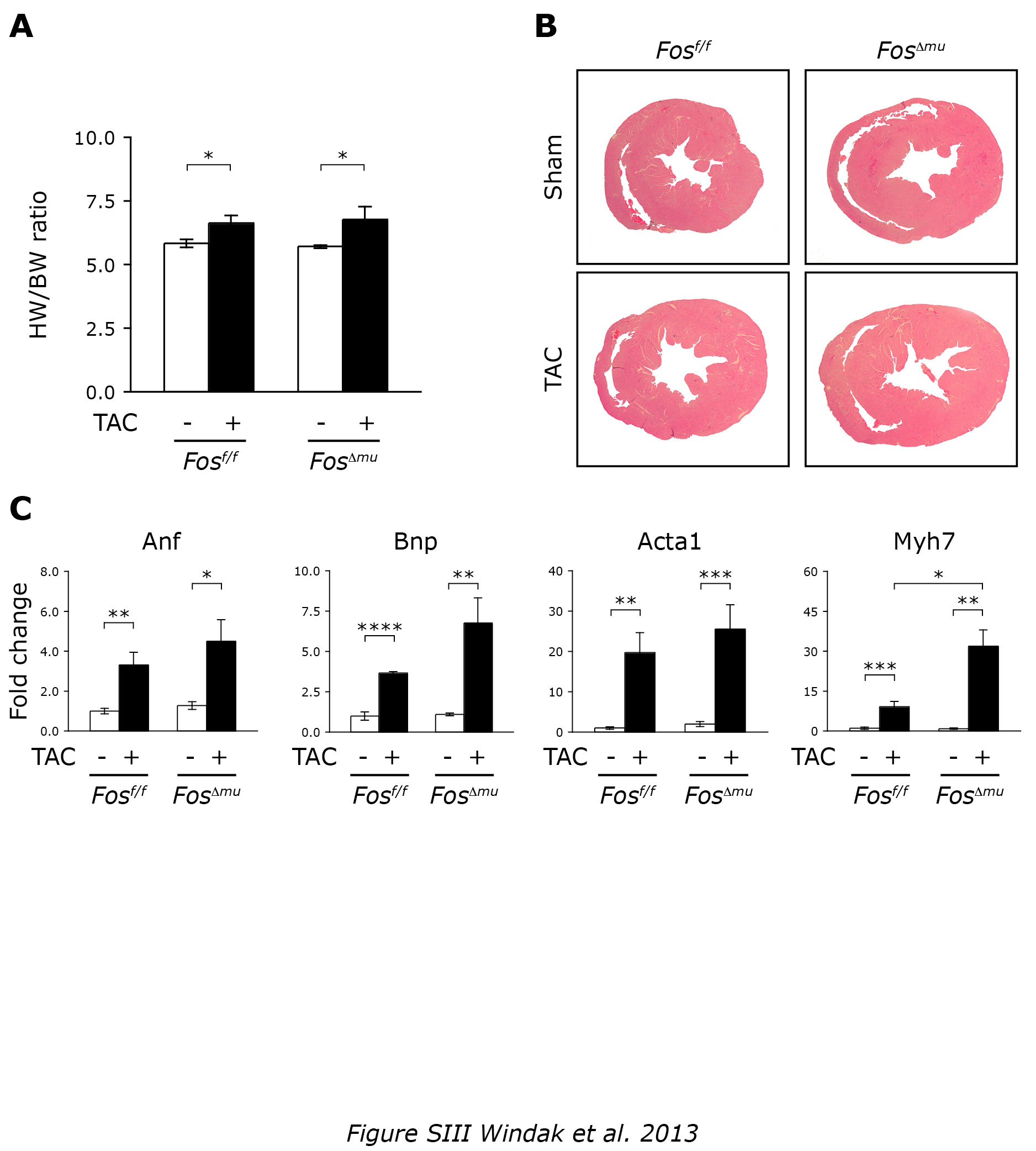

Supplement: Figure S3 — FosΔmu mice show concentric heart hypertrophy upon TAC. (A) H/BW ratio increases significantly in both Fosf/f and cFosΔmu mice upon TAC. Data are presented as values ± SEM. (*) p<0.05; n = 4–6 per group. (B) Histological analyses. H&E staining of heart cross-sections shows slight increase in left-ventricle wall thickness in both TAC-operated groups. (C) Relative expression of hypertrophic markers assessed by quantitative RT-PCR. Anf, Bnp, Acta1, and Myh7 are re-expressed in hypertrophied hearts of Fosf/f and FosΔmu mice. (*) p<0.05, (**) p<0.01, (***) p<0.001, (****) p<0.0001; n = 4–6 per group. (TIF) [file pone.0073294.s003.tif]

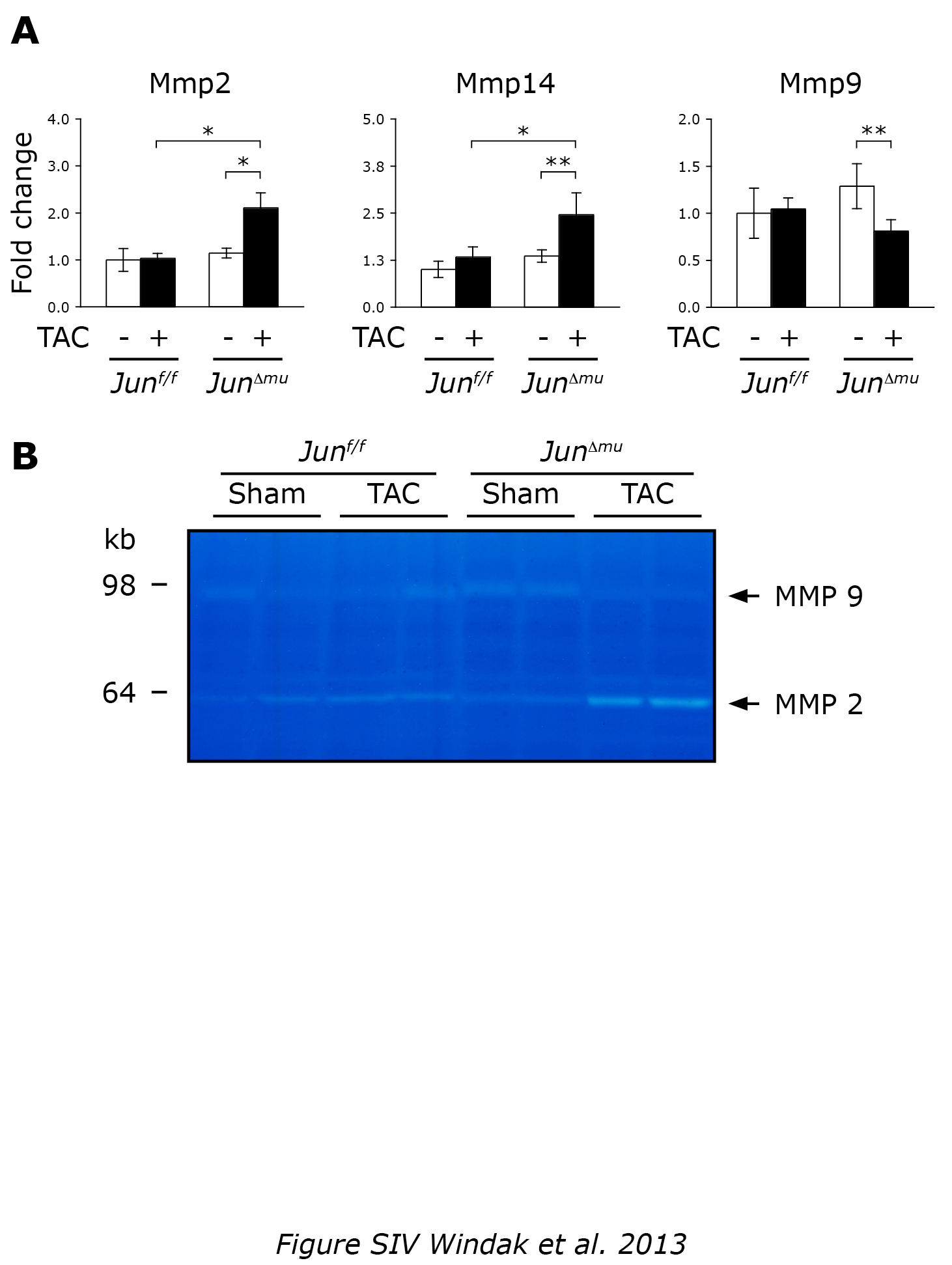

Supplement: Figure S4 — TAC-induced cardiac fibrosis in JunΔmu mice is associated with enhanced MMPs expression and activity. (A) Relative expression of myocardial MMPs (as indicated) assessed by quantitative RT-PCR, in sham and TAC operated mice from indicated genotypes. Data are presented as values ± SEM. (*) p<0.05, (**) p<0.01; n = 5 per group. (B) Gelatin zymography on total heart protein extracts. TAC-operated JunΔmu mice show greatly increased activity of MMP-2 (arrow at 66 kDa), and slightly decreased activity of MMP-9 (arrow at 92 kDa) in hearts as compared to sham-operated mice and TAC-operated Junf/f mice. No difference in MMP-2 and MMP-9 activity in hearts is observed between TAC operated Junf/f mice, when compared to sham-operated controls, as well as sham-operated Junf/f and JunΔmu mice. (TIF) [file pone.0073294.s004.tif]

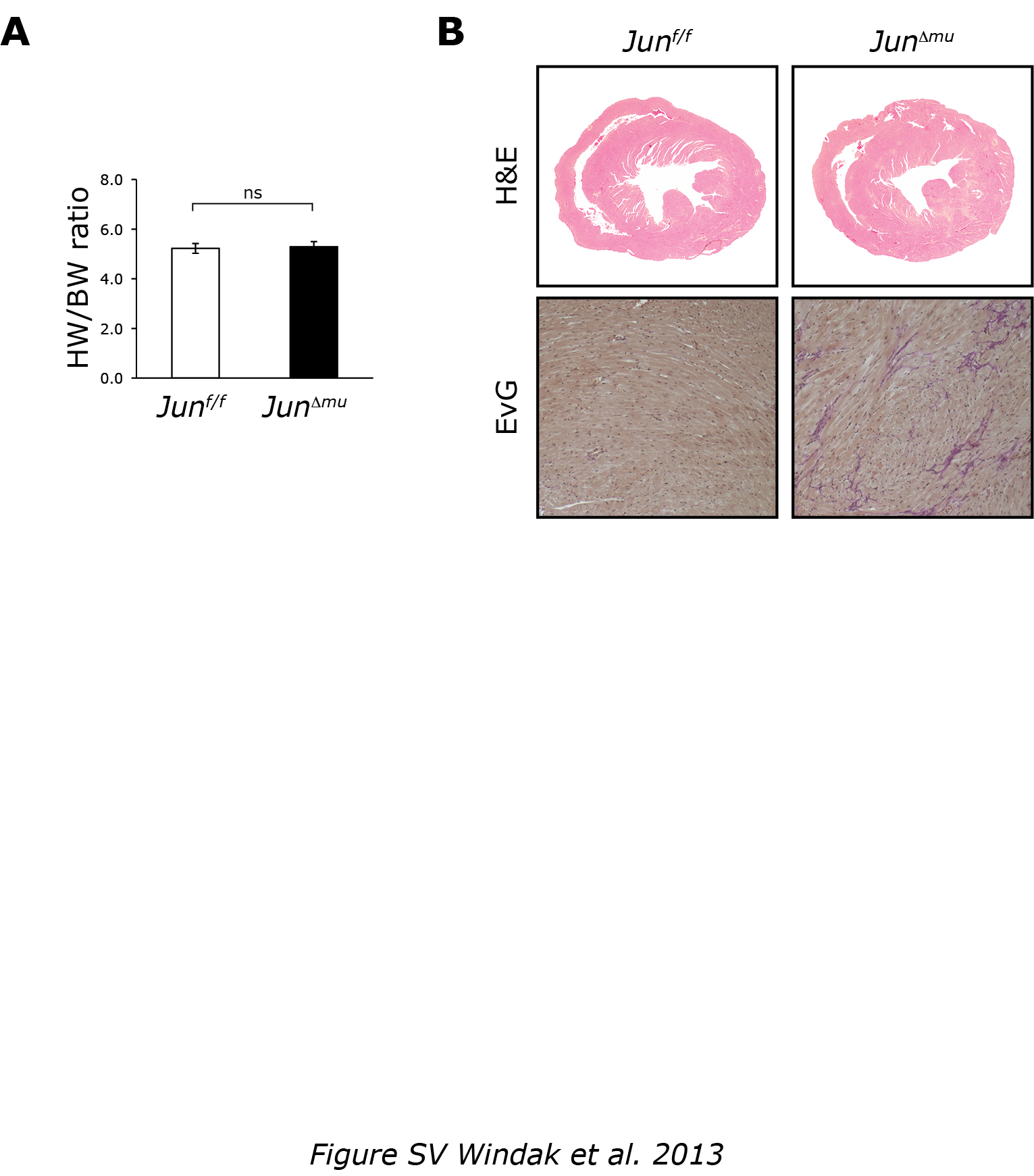

Supplement: Figure S5 — Hearts of one year old JunΔmu mice present spontaneous fibrosis. (A) Normal H/BW ratio of one year old JunΔmu mice. (B) Histological analyses. EvG staining reveals spontaneous fibrosis in hearts of one year old JunΔmu mice. (TIF) [file pone.0073294.s005.tif]
