# Supplementary material for: The AP-1 Transcription Factor c-Jun Prevents Stress-Imposed Maladaptive Remodeling of the Heart
Source: PLoS One. 2013 Sep 10;8(9):e73294. doi: 10.1371/journal.pone.0073294 (PMC3769267; doi:10.1371/journal.pone.0073294)
Supplement: Table S1 — Body and organs weights in adult Jun Δmu mice and corresponding control mice. (DOC) [file pone.0073294.s006.doc]

**Table S1. Body and organs weights in adult *Jun^Δmu^* mice and corresponding control mice.**

| Data measure | *Jun^f/f^* | *Jun^Δmu^* |
| --- | --- | --- |
| Body weight (g) | 25.9 ± 0.97 | 26.7 ± 0.78 |
| Heart weight (mg) | 134.8 ± 4.30 | 147.6 ± 8.55 |
| Liver weight (mg) | 1336.7 ± 82.2 | 1480.0 ± 97.9 |
| Lung weight (mg) | 154.5 ± 2.29 | 160.5 ± 5.77 |

All values are shown as mean ± SEM. WT n=6, KO n=8.
