# Supplementary material for: The AP-1 Transcription Factor c-Jun Prevents Stress-Imposed Maladaptive Remodeling of the Heart
Source: PLoS One. 2013 Sep 10;8(9):e73294. doi: 10.1371/journal.pone.0073294 (PMC3769267; doi:10.1371/journal.pone.0073294)
Supplement: Table S2 — Body and organs weights in adult Fos Δmu mice and corresponding control mice. (DOC) [file pone.0073294.s007.doc]

**Table S2. Body and organs weights in adult *Fos^Δmu^*  mice and corresponding control mice.**

| Data measure | *Fos^f/f^* | *Fos^Δmu^* |
| --- | --- | --- |
| Body weight (g) | 30.39 ± 0.94 | 30.52 ± 1.15 |
| Heart weight (mg) | 160.0 ± 9.55 | 145.0 ± 6.79 |
| Liver weight (mg) | 1284.6 ± 69.1 | 1287.2 ± 21.2 |
| Lung weight (mg) | 172.6 ± 4.98 | 162.4 ± 6.38 |

All values are shown as mean ± SEM. WT n=7, KO n=5.
