# Supplementary material for: The AP-1 Transcription Factor c-Jun Prevents Stress-Imposed Maladaptive Remodeling of the Heart
Source: PLoS One. 2013 Sep 10;8(9):e73294. doi: 10.1371/journal.pone.0073294 (PMC3769267; doi:10.1371/journal.pone.0073294)
Supplement: Table S3 — Echocardiographic analyses in Fos Δmu mice after TAC. (DOC) [file pone.0073294.s008.doc]

**Table S3. Echocardiographic analyses in *Fos^Δmu^* mice after TAC.**

|  | *Fos^f/f^* | | *Fos^Δmu^* | |
| --- | --- | --- | --- | --- |
| Data measure | sham | TAC | sham | TAC |
| HR, bpm | 492 ± 23 | 525 ± 38 | 456 ± 14 | 525 ± 32 |
| LVPWd, mm | 0.79 ± 0.019 | 0.92 ± 0.024 # | 0.79 ± 0.109 | 0.92 ± 0.011 † |
| LVPWs, mm | 0.99 ± 0.011 | 1.15 ± 0.020 # | 1.00 ± 0.021 | 1.14 ± 0.016 † |
| LVIDd, mm | 3.84 ± 0.075 | 3.84 ± 0.112 | 3.97 ± 0.056 | 3.73 ± 0.144 |
| LVIDs, mm | 2.78 ± 0.068 | 2.62 ± 0.107 | 2.88 ± 0.047 | 2.48 ± 0.141 † |
| FS, % | 27.52 ± 0.63 | 31.79 ± 0.94 # | 27.47 ± 0.68 | 33.66 ± 1.54 † |
| EF, % | 54.13 ± 1.02 | 60.52 ± 1.43 # | 53.93 ± 1.04 | 63.16 ± 2.22 † |

All values are shown as mean ± SE. n = 4-6 per group. p < 0.05 is indicated as: # WT TAC vs WT sham; † KO TAC vs KO sham; ¶ KO sham vs WT sham; § KO TAC vs WT TAC. HR, Heart rate; LVPWd, Left ventricular posterior wall in diastole; LVPWs, Left ventricular posterior wall in systole; LVIDd, Left ventricular internal diameter in diastole; LVIDs, Left ventricular internal diameter in systole; FS, Fractional Shortening; EF, Ejection Fraction.
