# Supplementary material for: The AP-1 Transcription Factor c-Jun Prevents Stress-Imposed Maladaptive Remodeling of the Heart
Source: PLoS One. 2013 Sep 10;8(9):e73294. doi: 10.1371/journal.pone.0073294 (PMC3769267; doi:10.1371/journal.pone.0073294)
Supplement: Table S4 — Echocardiographic assessment of heart function in one year old Jun Δmu and corresponding control mice. (DOC) [file pone.0073294.s009.doc]

**Table S4. Echocardiographic assessment of heart function in one year old *Jun^Δmu^*  and corresponding control mice.**

| Data measure | *c-jun^f/f^* | *c-jun^Δmu^* |
| --- | --- | --- |
| IVSd, mm | 0.57 ± 0.016 | 0.58 ± 0.014 |
| IVSs, mm | 0.66 ± 0.019 | 0.66 ± 0.021 |
| LVPWd, mm | 0.59 ± 0.023 | 0.56 ± 0.021 |
| LVPWs, mm | 0.76 ± 0.016 | 0.73 ± 0.039 |
| LVIDd, mm | 4.42 ± 0.076 | 4.38 ± 0.088 |
| LVIDs, mm | 3.46 ± 0.070 | 3.43 ± 0.106 |
| FS, % | 21.87 ± 0.41 | 21.74 ± 1.10 |
| EF, % | 44.43 ± 0.74 | 44.19 ± 1.95 |
| LV Mass, mg | 91.1 ± 5.15 | 88.50 ± 3.58 |
| LV VOLd, ml | 88.85 ± 3.56 | 86.73 ± 4.09 |
| LV VOLs, ml | 49.45 ± 2.38 | 48.61 ± 3.54 |
| LVID Trace (CO), ml/min | 18.56 ± 0.82 | 19.34 ± 0.67 |
| LVID Trace (SV), ml | 39.6 ± 1.69 | 38.9 ± 0.94 |

All values are shown as mean ± SEM. WT n=6, KO sham n=5. HR, Heart rate; IVSd, Interventricular septum in diastole; IVSs, Interventricular septum in systole; LVPWd, Left ventricular free wall in diastole; LVPWs, Left ventricular free wall in systole; LVIDd, Left ventricular internal diameter in diastole; LVIDs, Left ventricular free wall in systole; LV Mass, Left ventricular Mass; LV VOLd, Left ventricular Volume in diastole; LV VOLs, Left ventricular Volume in systole; FS, Fractional Shortening; EF, Ejection Fraction; LVID Trace (CO), LVID Trace (Cardiac output); LVID Trace (SV), LVID Trace (Stroke volume).
